# Partial left superior pulmonary vein potential elimination by an inferior ganglionated plexus ablation

**DOI:** 10.1002/ccr3.1188

**Published:** 2017-09-26

**Authors:** Yasutsugu Nagamoto, Yuto Fujii, Yuichi Morita, Yusuke Ueda, Kenichi Yamane, Yasuko Miyake, Mai Fujiwara, Shinji Mito, Yuichiro Watari, Hiromichi Tamekiyo, Tomokazu Okimoto, Yuji Muraoka, Yasuhiko Hayashi

**Affiliations:** ^1^ Division of Cardiology Tsuchiya general hospital Hiroshima Japan

**Keywords:** Ganglionated plexus, pulmonary vein potentials

## Abstract

Ganglionated plexus (GP) plays an important role in the initiation and maintenance of atrial fibrillation (AF). The GP ablation has been found to be effective for AF treatment. In this case, we reported an AF case in which the pulmonary vein (PV) potentials of the anterior region of the left superior PV were eliminated by an inferior right GP ablation.

## Case Report

A 71‐year‐old man with hypertension underwent a cavotricuspid isthmus (CTI) ablation for a CTI‐dependent atrial flutter (AFL) at the age of 62 years . Drug resistance paroxysmal atrial fibrillation (AF) occurred after the AFL ablation, and therefore, an AF ablation was performed under the use of three‐dimensional electroanatomical mapping (NavX System, St. Jude Medical Inc., St. Paul, MN, USA). Ultrasound echocardiography revealed no structural heart disease and a left atrial (LA) dimension of 34 mm. The LA volume calculated by computed tomography was 72 cm^3^. Ganglionated plexus (GP) clusters were identified as sites where vagal reflexes were evoked by transcatheter high‐frequency stimulation. Regarding the GP ablation, radiofrequency (RF) applications for a circumferential pulmonary vein (PV) isolation were performed to cover the superior left and anterior right GP sites. Inferior GP sites were observed far from the circumferential PV isolation line. Therefore, RF applications to the inferior GP were performed separately from the PV isolation line.

**Figure 1:**
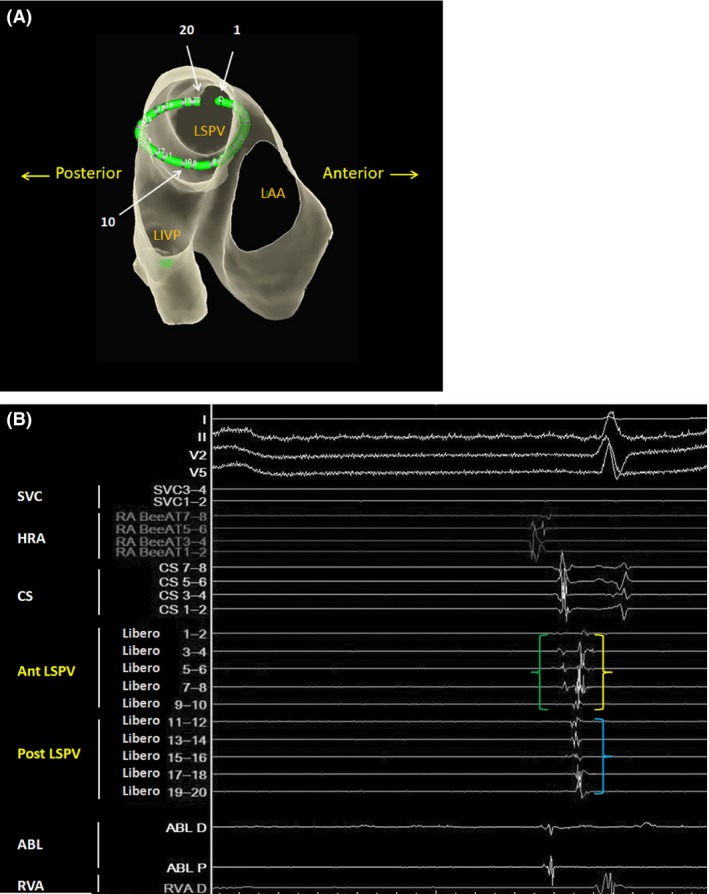
(A) Transparent view from the inside of the left atrium to the left lateral side. The green ring catheter is placed at the LSPV. Electrodes 1–10 on the ring catheter are located at the anterior part of the LSPV, while 11–20 cover the posterior part of the LSPV. LAA, left atrial appendage; LIPV, left inferior pulmonary vein; LSPV, left superior pulmonary vein (B) Intracardiac ECG showing the pre‐ablation LSPV potentials. The first potential from the anterior LSPV (green bracket) is the far‐field potential from the left atrial appendage. The second potential recorded from Libero electrodes 1–2 to 9–10 is from the anterior part of the LSPV (yellow bracket). The potential from Libero electrodes 11–12 to 19–20 exhibits the potentials from the posterior part of the LSPV (blue bracket) ABL, ablation catheter; Ant LSPV, anterior part of the left superior pulmonary vein; CS, coronary sinus; HRA, high right atrium; Post LSPV, posterior part of the left superior pulmonary vein; RVA, right ventricular apex; SVC, superior vena cava.

**Figure 2:**
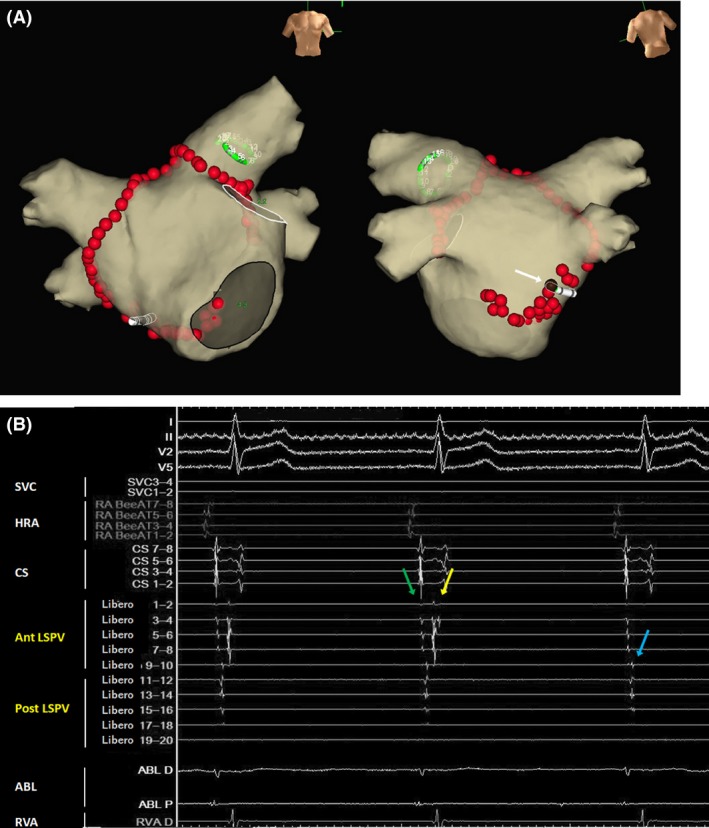
(A) RF application sites at the anterior part of the left PV, roof, anterior part of the right PV, and inferior GP ablation. The potentials from the anterior part of the LSPV are eliminated by the RF application at the inferior right GP (brown dot [white arrow]). The green ring catheter at the LSPV and white ablation catheter at the RF application site are displayed. GP, ganglionated plexus; LSPV, left superior pulmonary vein; PV, pulmonary vein; RF, radiofrequency (B) Intracardiac ECG showing the elimination of the PV potentials of the anterior part of the LSPV (yellow arrow). The potentials from the posterior part of the LSPV are unchanged (blue arrow). The first potential from the anterior LSPV (green arrow) is the far‐field potential from the left atrial appendage. Abbreviations are listed in Figure 1B.

**Figure 3:**
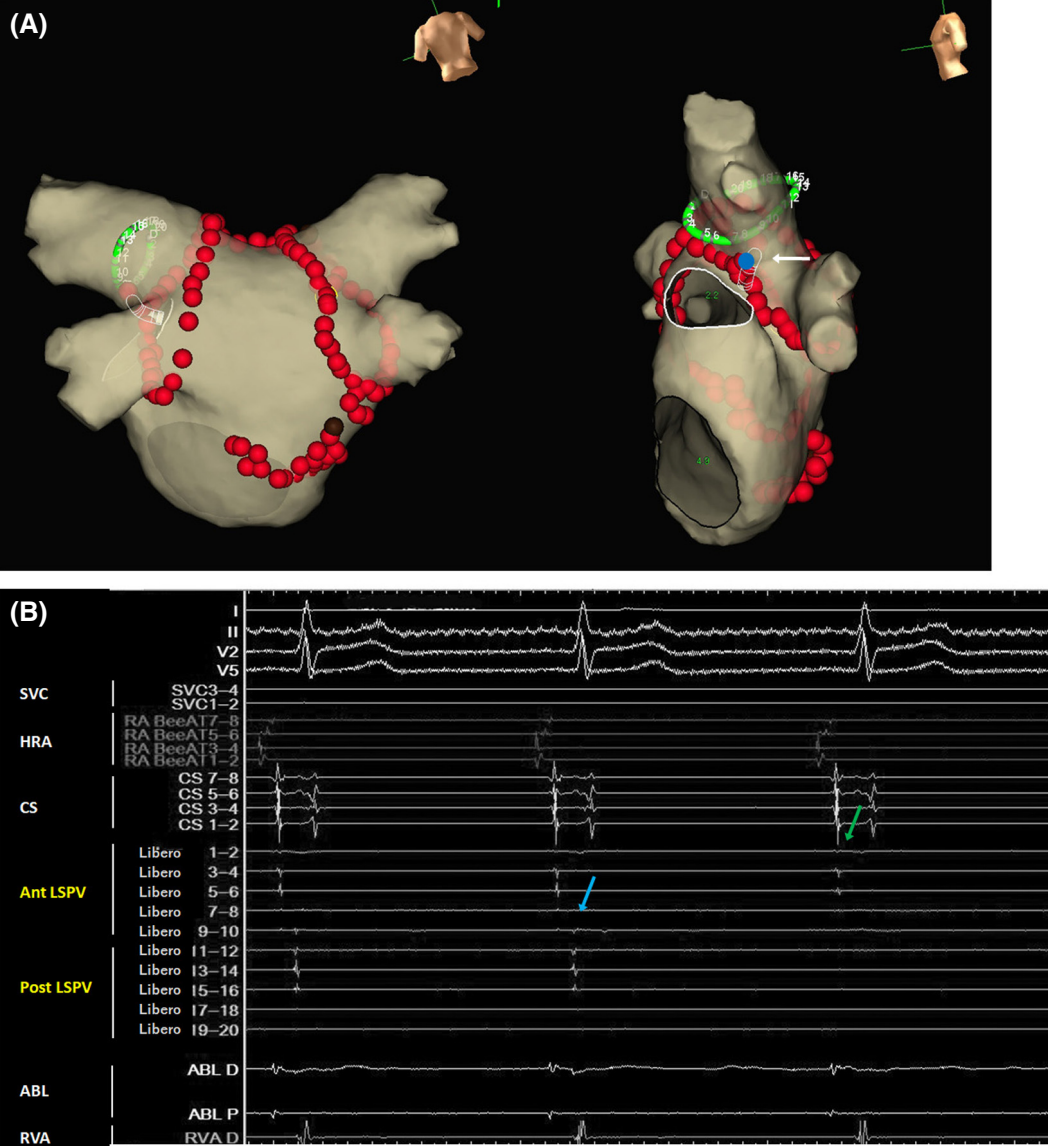
(A) Total RF application sites during the AF ablation. The potentials from the posterior part of the LSPV were eliminated by the RF application at the anterior carina of the left PVs, which lead to a left PV isolation (blue dot [white arrow]). The green ring catheter at the LSPV and silhouette‐shaped ablation catheter at the RF application site are displayed. The abbreviations are listed in Figure 2A. (B) Intracardiac ECG showing the elimination of the potentials from the posterior part of the LSPV (blue arrow). The potentials from the anterior LSPV (green arrow) are the far‐field potentials from the left atrial appendage. The abbreviations are listed in Figure 1B.

Radiofrequency applications with a 7.5Fr, 3.5‐mm‐tip‐irrigated ablation catheter (FlexAbility^™^, St. Jude Medical Inc., St. Paul, MN, USA) were performed at the anterior part of the left superior and inferior PVs, roof, anterior part of the right superior and inferior PVs, and site of the inferior left GP in that order. A 20‐pole ring catheter with a 17.5 mm size (EP star Libero, Japan Lifeline Co., Tokyo, Japan) placed at the left superior PV (LSPV) and intracardiac electrocardiogram (ECG) showing the pre‐ablation LSPV potentials are shown in Figure 1. The PV potentials recorded by the Libero electrodes 7–8 were delayed during the RF applications above. An RF application to the inferior right GP eliminated the PV potentials from the anterior part of the LSPV while the PV potentials from the posterior part of the LSPV were unchanged (Fig. 2). The PV potentials recorded from the Libero electrodes 7–8 were delayed before the RF application at the inferior GP, and the sequence of the PV potentials at the anterior LSPV was almost the same as that of the pre‐ablation PV potentials (Fig. 2B). Therefore, those PV potentials recorded from Libero electrodes 1–8 were estimated to represent the delayed residual anterior potentials, and not the potentials from the posterior wall. The RF application to the anterior carina of the left PVs eliminated the PV potentials of the posterior part of the LSPV, which led to a left PV isolation (Fig. 3), and exit block was confirmed by PV pacing from a ring catheter. Finally, RF applications to the posterior part of both PVs were delivered. The AF ablation was completed successfully by a circumferential PV isolation, linear roof ablation, and inferior left and right GP ablation, which are shown in Figure 3A.

## Discussion

The initiation and maintenance of AF is caused by intrinsic cardiac autonomic activity 1; furthermore, PV firing can be initiated by GP stimulation 2. GP ablation has been found to be effective for AF treatment, by targeting the anatomic sites or sites where vagal reflexes are evoked by high‐frequency stimulation 3. A previous study demonstrated that a GP ablation eliminated rapid firing in an electrically isolated PV 4. Thus, GP ablation has been reported to be effective for eliminating the initiation and maintenance of AF 5. However, to the best of our knowledge, this is the first report to demonstrate the elimination of a connection between a PV and the LA by a GP ablation.

Vagal nerve stimulation has been reported to shorten the atrial effective refractory period (AERP) and increase the AERP heterogeneity 1. Adrenergic and cholinergic nerve densities are the highest in the LA within 5 mm of the PV–LA junction 6. The PV potentials at the anterior part of the LSPV were eliminated by an inferior right GP ablation. When evaluating the effect of RF applications, a delayed effect should be considered. In this patient, the RF applications were started at the anterior LSPV, and the time interval between the direct RF application and elimination of the PV potentials at the anterior part of the LSPV was more than 30 min. Therefore, a delayed effect of a direct RF application was unlikely. Furthermore, an RF application at the inferior right GP alone has a high possibility of not eliminating the PV potentials in this part. The phenomenon in this patient was a cumulative effect of the RF applications. First, the RF application to the anterior part of the LSPV directly destabilized the conduction of the anterior part of the LSPV. Then, the inferior right GP ablation might have had an impact on the effective refractory period of the anterior part of the LSPV, which indirectly resulted in the elimination of the PV potentials in that part. In conclusion, the inferior GP ablation itself had no efficacy of eliminating the PV potentials; however, it might have had an additional impact of eliminating the PV potentials by a cumulative effect followed by a direct RF application at the PVs.

## Authorship

YF, YM, YU, KY, YM, MF, SM, YW, HT, TO, YM, YH: are the members at out division of cardiology.

## Conflict of Interest Disclosures

None.
